# Apoptotic Effects of γ-Mangostin from the Fruit Hull of *Garcinia mangostana* on Human Malignant Glioma Cells

**DOI:** 10.3390/molecules15128953

**Published:** 2010-12-07

**Authors:** Hui-Fang Chang, Wen-Tsung Huang, Hui-Ju Chen, Ling-Ling Yang

**Affiliations:** 1 Department of Pharmacognosy, School of Pharmacy, College of Pharmacy, and Center of e-CAM, Taipei Medical University, 250 Wusing St., Taipei 110, Taiwan; E-Mail: beautymoon16@yahoo.com.tw (H-F.C.); 2 Division of Hemato-Oncology, Chi-Mei Medical Center, 201 Taikang Village, Liouying Township, Tainan County 736, Taiwan; E-Mail: huang570725@gmail.com (W-T.H.); 3 Center of Translational Research on Traditional Medicine, Institute of Clinical Medical Science, China Medical University and Hospital, 2 Yuh-Der Road, Taichung 40447, Taiwan; E-Mail: chenhj@mail.cmuh.org.tw (H-J.C.)

**Keywords:** γ-mangostin, high-grade brain tumor, antiproliferation, apoptosis, reactive oxygen species

## Abstract

Gliomas are a common type of primary brain tumor with glioblastoma multiforme accounting for the majority of human brain tumors. In this paper, high grade human malignant glioblastomas (MGs) including U87 MG and GBM 8401 were used to evaluate the antitumor effects of γ-mangostin, a xanthone derivative isolated and purified from the hull of the tropical fruit *Garcinia mangostana*. The γ-mangostin showed potent antiproliferative activity toward MGs in dose- and time-dependent manners. In addition, flow cytometric analysis of cell morphology in the apoptotic cells revealed an increase in hypodiploid cells in γ-mangostin treated U87 MG and GBM 8401 cells, while significant enhancement of intracellular peroxide production was detected in the same γ-mangostin treated cells by DCHDA assay and DiOC_6_(3) stain. γ-Mangostin induced apoptosis, which in turn mediates cytotoxicity in human MG cells was prevented by the addition of catalase. Naturally derived medicines and herbal therapies are drawing increasing attention in regard to the treatment of many health issues, and this includes the testing of new phytochemicals or nutrients for brain tumor patients. This has led to γ-mangostin being identified as a potential leading compound for the development of an anti-brain tumor agent.

## 1. Introduction

The xanthone derivatives, mangostins, are derived from mangosteen, *Garcinia mangostana* Linn. Mangosteen, known as ‘the queen of fruit’, is a tropical, fragrant fruit with a sweet tangy flavor, and in Thailand, the pericarp has long been valued as an ingredient in traditional medicines [1]. Over the past ten years, the antitumorigenic effect and action mechanism of γ-mangostin have been investigated and potent antitumorigenic lead compounds have been reported. They are α-, β-, γ−, and methoxy-β-mangostin, with the latter differing in the number of hydroxyl and methoxy groups. Alpha-, β- and γ-mangostin have been reported to strongly inhibit cell proliferation in human colon cancer DLD-1 cells [2] and exert antimetastatic activity in mouse metastatic mammary cancer models [3]. 

**Figure 1:**
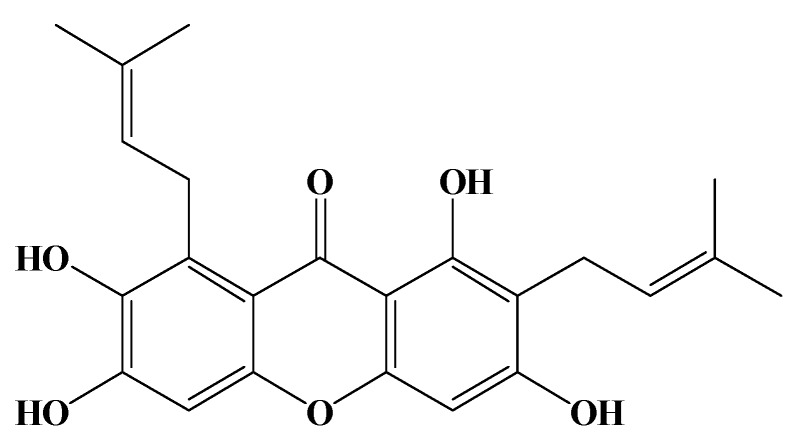
Structure of γ-mangostin.

The efficacy of chemotherapy in the treatment of high grade glial neoplasms has been generally disappointing. Approximately 17,000 primary brain tumors are diagnosed every year in the US, and of those, about 60% are gliomas. The median survival rate is less than a year from diagnosis, and this rate has remained relatively constant over the past 25 years [4]. Over the last 10 years, only two chemotherapeutic agents, carmustine (1,3-bis(2-chloroethyl)-1-nitrosourea, BCNU implant) and temozolomide (TMZ), an imidazotetrazine derivative of dacarbazine, have received regulatory approval for treating malignant gliomas [5]. High doses of BCNU are visceral toxic and cause extensive myelosuppression [6]. Clearly, new therapies are required, though the basis for their development still lies in building on the technical strengths of current ones. Phytochemicals, or phytonutrients, are naturally occurring chemical compounds in plants that are known to be beneficial to human health, and as such, the examination of potential botanical therapies has become a new frontier for research into cancer treatment [7]. γ-Mangostin, a tetraoxygenated diprenylated xanthone derivative, is one such phytochemical molecule (Figure 1).

For this study, over 100 kinds of natural polyphenols were isolated from Taiwanese folk medicines and examined in regard to both high grade U87 MG and GBM 8401 brain tumor cells with γ-mangostin being identified as one of the strongest antiproliferative compounds. In a previous study comparing α- and γ-mangostin, we had detected anti-inflammatory activity but no inhibitory ability [8]. This study was designed to determine γ-mangostin’s antioxidative and antiproliferative status, and the mechanism of the cytotoxic effect it exerts. The γ-mangostin was extracted from the pericarp of *Garcinia mangostana* (family Guttiferae). While malignant glioblastomas also represent the most common type of primary brain tumor in adults, given the dearth of efficient therapeutic treatments for these highly malignant gliomas, Chinese traditional medicines and ethnobotany are increasingly being identified by researchers as promising bioresources for developing anti-brain tumor agents, while many traditional Chinese medicines are already being used by local cancer patients in Taiwan. Recently, two papers reported on the development of brain-tumor drugs from natural sources, they are danggui and triptolide [9,10]. In our study, the human MG cell lines of an astrocytoma glioblastoma, grade III-U87 MG, and glioblastoma multiforme, grade IV-GBM 8410 were used as target cells.

## 2. Results and Discussion

### 2.1. Antiproliferation of γ-mangostin in U87 MG and GBM 8401 glioma cells

U87 MG (astrocytoma glioblastoma, grade III) and GBM 8401 (glastomaliob multiforme, grade IV) were used to measure the rates of cell proliferation and cell death in models of both human MG cell lines. An apparent antiproliferative ability of γ-mangostin on human U87 MG and GBM 8401 cells was exhibited after drug treatment for 24 hours by an MTT assay (Table 1). 

**Table 1:** Cytotoxicity of γ-mangostin in human glioblastoma cells. Cells treated with different doses of γ-mangostin for 24 hours. Cytotoxicity determined by MTT assay (method described in Experimental).

Drug (μM)	Cytotoxicity (%)	
	U87 MG	GBM 8401
γ-mangostin		
10	0.00 ± 9.01	0.00 ± 0.00
20	0.00 ± 4.79	11.97 ± 3.12
40	3.64 ± 5.28	22.79 ± 6.20
80	55.76 ± 7.38	65.58 ± 0.43
100	89.18 ± 3.79	76.18 ± 7.14
200	98.83 ± 0.27	99.34 ± 0.13
BCNU^a^		
934	71.47 ± 5.49	92.30 ± 2.50

^a^ BCNU, carmustine, [1,3-bis(2-chloroethyl)-1-nitrosourea]. It is a brain tumor chemotherapy agent which is a positive control on both U87 MG and GBM 8401 cell lines for 24 hours treatment, respectively. Each concentration repeated three times (mean ± SD).

The γ-mangostin reduced the viability of both U87 MG and GBM 8401 cells in a dose-dependent manner. Within 24 hours, a significant, rapid decrease of more than fifty percent of viable U87 MG and GBM 8401 cells was recorded with IC_50_ doses of γ-mangostin (Table 2). The IC_50_ values of γ-mangostin were 74.14 ± 2.93 and 64.67 ± 2.42 μM; a 5 ~ 8 fold superiority to that of the control drug, carmustine (BCNU), whose IC_50_ values were 632.1 and 346.6 μM in U87 MG and GBM 8401 cells, respectively. The γ-mangostin inhibited the cell proliferation in dose- and time-dependent modes for both cell types.

**Table 2:** The time dependent cytotoxicity of γ-mangostin in human glioblastoma cells. Brain cancer cells treated for different times (24, 48 and 72 hours) with IC_50_ concentration **γ**-mangostin. Cell cytotoxicity was analyzed by MTT assay.

Cell line	IC_50_ (μM)^a^	Cytotoxicity (%)		
		24 h	48 h	72 h
U87 MG	74.14 ± 2.93	59.89 ± 0.03	73.78 ± 0.30	90.25 ± 2.71
GBM 8401	64.67 ± 2.42	54.22 ± 0.04	60.92 ± 0.01	99.08 ± 0.36

^a^ BCNU, its IC_50_ values were 632.11 and 346.65 μM on both U87 MG and GBM 8401 cell lines for 24 hours treatment, respectively. Each concentration repeated three times (mean ± SD)

### 2.2. γ-Mangostin induces the apoptosis process via intracellular ROS production

**Figure 2:**
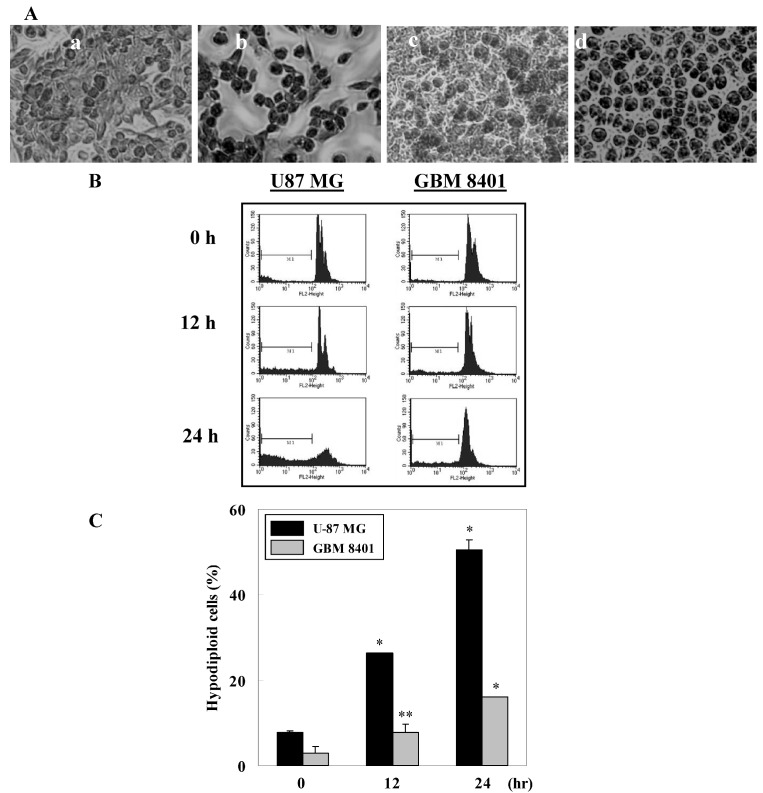
Morphological changes and hypodiploid cells in γ-mangostin treated U87 MG and GBM 8401 cells. (A) U87 MG (a, b) and GBM 8401 (c, d) cells were treated with (b, d) or without (a, c) γ-mangostin (80 μM) for 24 hours followed by Giemsa staining, and condensed cells (b,d) were observed under a 200 fold zoom-in microscope. (B) Appearance of hypodiploid cells in γ-mangostin treated U87 MG and GBM 8401 cells were shown by PI stain. Cells were treated with γ-mangostin (80 μM) for 8, 12, and 24 h, the sub-G1 (M1, hypodiploid) region was detected and quantified by flow cytometry. (C) Quantitative analysis of the ratio of hypodiploid cells in cells subjected to γ-mangostin (80 μM) treatment for the indicated time points. Data are presented as the mean ± SD of three independent experiments. *p < 0.05 and **p < 0.01 indicate significant differences from the control group (CTL) as analyzed by Student’s *t*-test.

Microscopic observations showed nuclear condensation in γ-mangostin treated cells via Giemsa staining (Figure 2A). The ratio of hypodiploid cells (sub-G1 peak) in γ-mangostin treated cells significantly increased according to a flow cytometric analysis via PI staining (Figure 2B). A quantification of the sub-G1 regions (label on M1 region) relative to different time periods revealed a more sensitive apoptosis level of U87 MG cells compared to GBM8401 (Figure 2C). A changeable morphology and an increase in the sub-G1 region suggest that the cytotoxic activity in human glioblastoma cells is a result of γ-mangostin induced apoptosis. 

**Figure 3:**
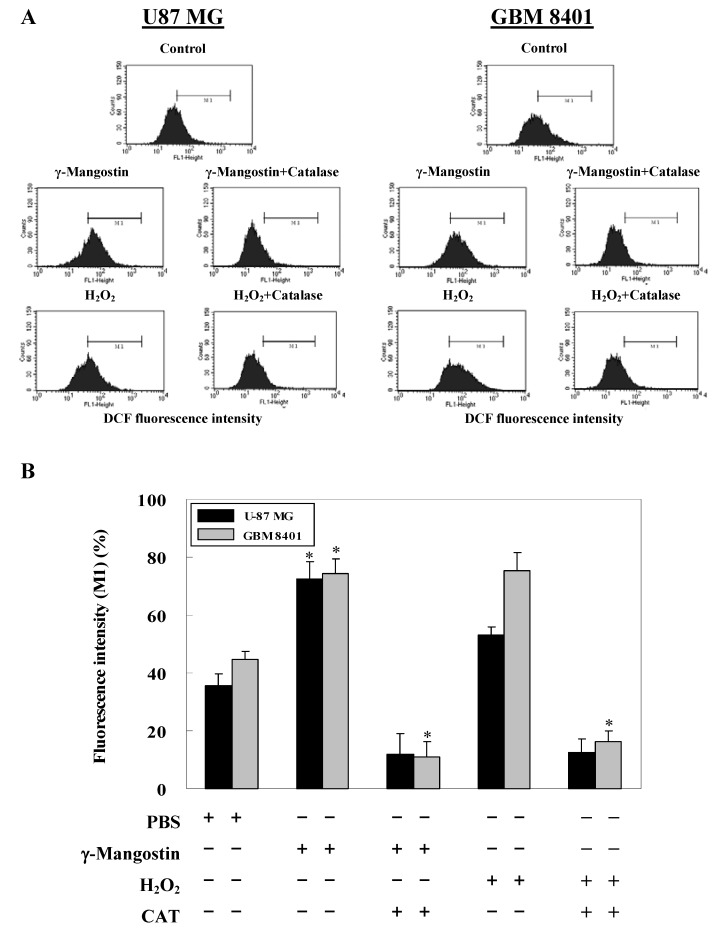
Increased intracellular peroxide levels in γ-mangostin treated U87 MG and GBM 8401 cells. (A) Cells were treated with γ-mangostin (80 μM) or H_2_O_2_ (1 mM) for 1 hour with or without prior treatment with catalase (CAT; 400 U / mL) for 30 min. At the end of the reaction, the level of intracellular peroxide was examined by adding DCHDA for an additional 30 min followed by a flow cytometric analysis. (B) γ-Mangostin induced the intracellular peroxide, which was measured quantitatively through the intensity of DCFDA (DCF fluorescent intensity), by 50% compared to the untreated cells region (M1) of the control group on both cell lines. Data derived from three independent experiments were calculated statistically, and results are presented as the mean ± SD. * *p* < 0.05 and ** *p* < 0.01 indicate significant differences from the control group as analyzed by Student’s *t*-test.

Data on DCFDA staining also shows that γ-mangostin treatment increased the more intracellular peroxide level to H_2_O_2_ induced system. We compare to γ-mangostin (80 μM) with H_2_O_2_ (1 mM) induced system that both induced about 70% intracellular peroxide production on GBM 8401 cells, but H_2_O_2_ induced system revealed less intracellular peroxide levels which only induced about 50% intracellular peroxide production on U87 MG cells (Figure 3A). Quantitative data shown in Figure 3B indicate a concurrent reduction of intracellular peroxide induced by either γ-mangostin or H_2_O_2_ was due to the additional CAT added. The DCFDA data correspond to previous apoptosis morphology observation results γ-mangostin may induce glioma cell cytotoxity via ROS production.

**Figure 4:**
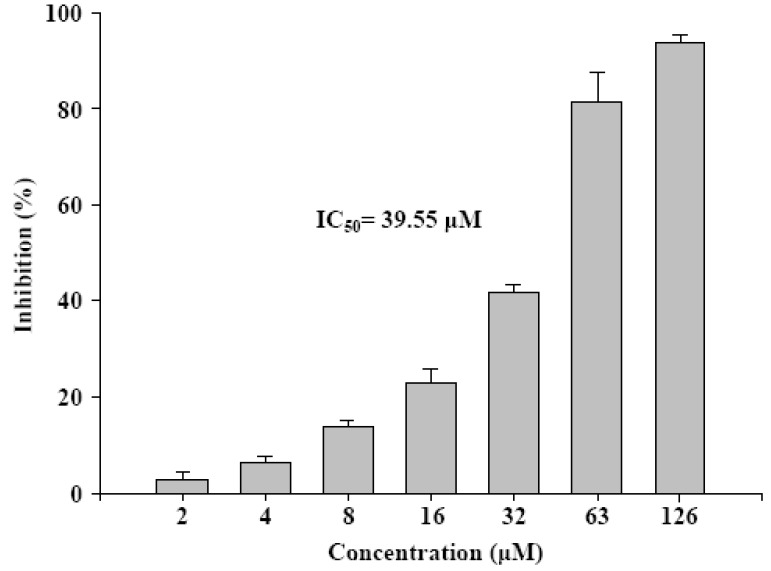
γ-Mangostin shows a dose dependent inhibitory activity on Fe^2+^-induced lipid peroxidation in rat brain. The homogenate brain mitochondrial were treated with γ-mangostin and MDA production was detected by TBARS assay.

MDA(TBA)_2 _produced was measured by the TBARS method (Figure 4). In Thailand, the rind of mangosteen, *G. mangostana* has been used for centuries to treat trauma, diarrhea, and skin infections [11], and one of its major components is γ-mangostin, which is known to participate in inhibiting 5-hydroxytryptamine (2A) receptors in the CNS [11], possess anti-inflammatory properties [12,13,14], and induce cell cycle arrest and apoptosis in human colon cancer DLD-1 cells [2]. Furthermore, it can inhibit breast cancer by aromatase inactivation [15], decrease human immunodeficiency virus (HIV) infection [16], and even function as histaminergic and serotonergic receptor-blocking agents [17]. It has also been demonstrated that γ-mangostin is a stronger antioxidant than vitamin E, one of the most powerful antioxidants known to science. 

Antioxidant activities were determined using authentic and morpholinosydnonimine-derived peroxynitrite methods, and γ-mangostin was identified as being the most active [18]. γ-Mangostin exhibited enhancement of NK cell activity in a mouse model, and it decreased the level of prostaglandin E2 (PGE2) through inhibition of cyclooxygenase (COX-2) activity and NO production. These findings provide a relevant basis for the development of xanthones as agents for cancer prevention and combination therapy with anticancer drugs [19].

### 2.3. Cell damage and ROS-dependent mitochondrial dysfunction

**Figure 5:**
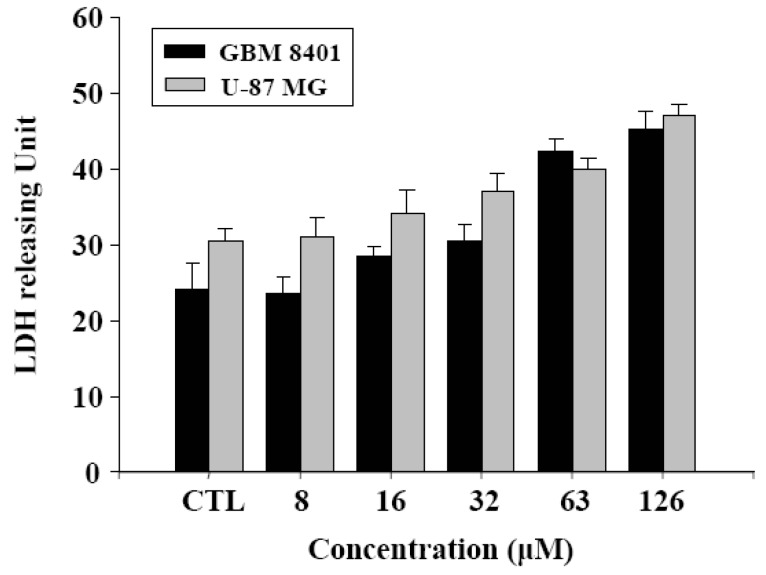
LDH releasing unit of γ-mangostin treatment in human glioblastoma cells. To evaluate the different doses of γ-mangostin, cell damage was observed for 24 hours on glioblastoma cell lines. The LDH activity in the supernatant of cells was determined by an indirect colorimetric assay. One unit of LDH released represents the generation of 1 μmol NADH through the reduction of 1 μmol L-lactate and then generated NADH which conversed and depended on 2-[4-iodophenyl]-5-phenyltetrazolium chloride (INT) by diaphorase at room temperature [20].

**Figure 6:**
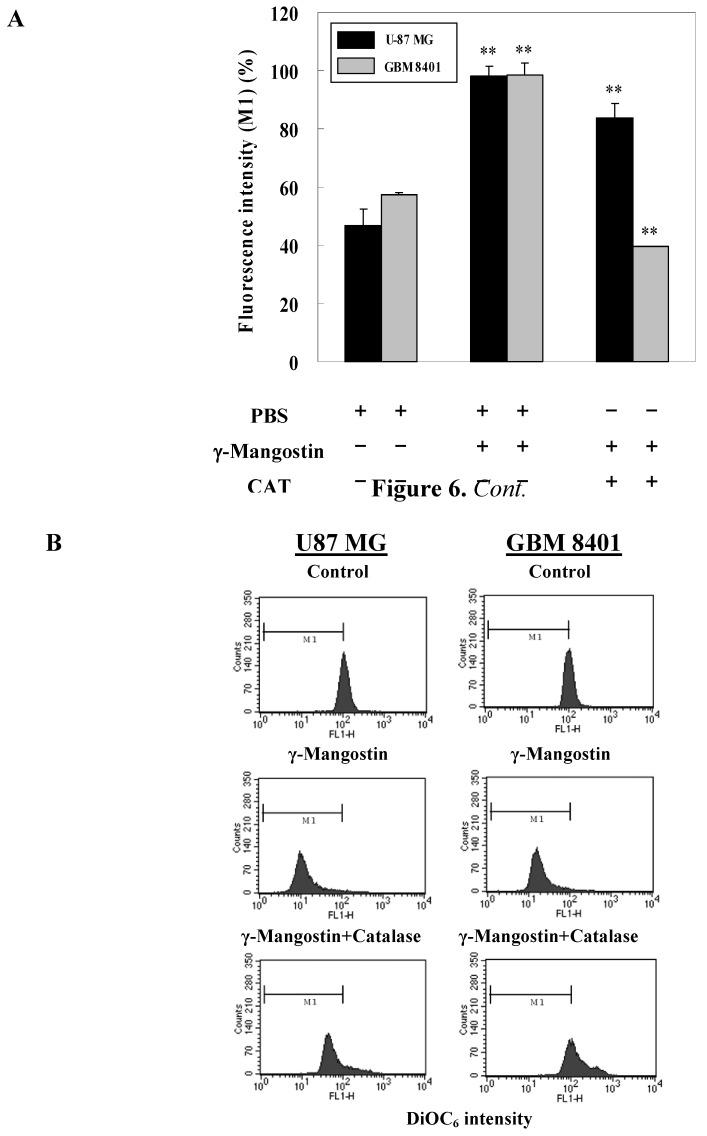
Catalase (CAT) prevented potential γ-mangostin-induced mitochondrial membrane dysfunction. (A) Cells were treated with γ-mangostin (80 μM) for 1 hour with or without prior treatment with CAT (400 U / mL) or PBS for 3 hours. At the end of the incubation, the mitochondrial membrane potential was analyzed by adding DiOC_6_(3) as a fluorescent substrate on flow cytometric analysis. (B) To quantity data of the M1 region which is a half of control group as well as γ-mangostin induced mitochondrial dysfunction was derived from three independent experiments, and those results were presented as the mean ± SD. * *p* < 0.05 and ** *p* < 0.01 indicate significant differences from the control group as analyzed by Student’s *t*-test.

Cell damage status was examined in order to gain a functional understanding of γ-mangostin activity. An increase in the LDH release of human glioblastoma cells concurred with a raised dosage of γ-mangostin (Figure 5). γ-Mangostin-induced mitochondrial dysfunction was monitored by the strength of fluorescent intensity of DiOC_6_(3) shown in the data of the flow cytometric analysis in U87 MG and GBM 8401 cells. Quantitative data was shown in Figure 6B indicate that mitochondrial dysfunction induced by γ-mangostin was reduced by the addition of CAT, which GBM 8401 cell was combined with additional CAT that was able to significantly block 60% cells from undergoing γ-mangostin induced mitochondrial dysfunction, but 10% on U87 MG cell (Figure 6B). Interestingly, a DCHDA assay revealed an increased amount of intracellular peroxide production in γ-mangostin treated U87 MG and GBM 8401 cells (Figure 3). γ-Mangostin produced a cytotoxic effect on MG cells, especially in relation to intracellular LDH release and mitochondrial dysfunction (Figure 5 & Figure 6). On the astrocytoma glioblastoma, grade III-U87 MG, a numerous and effective intracellular reactive oxygen species (ROS) maybe kill it and clear the cells by mediated more than H_2_O_2_ species. U87 MG cells revealed the effective tendency on sub-G1 peak production, and it also shown a resistant on CAT converted the function of damaged mitochondrial membrane (Figure 6B). In addition, there appeared to be a therapeutic relation to ROS induction, and the development of a strategy to improve malignant glioblastoma treatment might incorporate this feature.

## 3. Experimental

### 3.1. Chemicals

3-(4,5-Dimethylthiazol-2-yl)-2,5-diphenyltetrazolium bromide (MTT), and dimethyl sulfoxide (DMSO) were purchased from Sigma. Alpha minimum essential medium (α-MEM), fetal bovine serum (FBS), L-glutamine, and penicillin-streptomycin were purchased from Gibco. All chemical solvents were analytical grade and purchased from Merck. The propidium iodide (PI) staining solution was purchased from BD. The 2’7’-dichlorofluorescein diacetate (DCFDA) fluorescent dye and 3,3′-dihexyloxacarbocyanine iodide (DiOC_6_(3)) were purchased from Invitrogen.

### 3.2. Extraction, isolation, and purification of γ-mangostin

The fruit of *G. mangostana* was purchased from a fruit market in Taipei, Taiwan. The procedures of extraction, separation, isolation, purification, and structure determination have been described in a previous report [8]. In brief, fresh fruit hulls of *G. mangostana* were homogenized with 70% acetone. The extract was filtered and concentrated by a rotary evaporator to remove the acetone, and a reddish-brown extract was obtained. The extract was dissolved in ethyl acetate (EtOAc) and filtered; the filtrate was coated on Celite 545, and then subjected to silica gel column chromatography with a gradient elution system of *n*-hexane-EtOAc. The concentrated *n*-hexane-EtOAc eluate solution was rechromatographed through a silica gel column and eluted with a CHCl_3_-MeOH gradient to afford γ-mangostin as a fine yellow powder, C_23_H_24_O_6_, with a molecular weight of 396.43. Its purity was determined by reverse-phase high performance liquid chromatography (HPLC) and was shown to exceed 98.5%. The structure of γ-mangostin, previously determined by Chen *et al*. [8] is shown in Figure 1. A test solution of γ-mangostin (10 mM) was prepared by dissolving it in DMSO as a stock solution, whereupon it was stored at 4 °C until used. Serial dilutions of the test solutions with culture medium were prepared immediately before each assay was performed.

### 3.3. Cell lines and cell culture

The human malignant glioblastoma cell lines, U87 MG (astrocytoma glioblastoma, grade III) and GBM 8401 (glastomaliob multiforme, grade IV), were obtained from the Bioresource Collection and Research Center (BCRC). U87 MG and GBM 8401 cells were grown in 90% Eagle's minimum essential medium/RPMI 1640 medium supplemented with 10% fetal bovine serum, 1.5 mM L-glutamine, 100 units/mL penicillin, and 100 μg/mL streptomycin. All cell cultures were incubated at 37 °C in a humidified atmosphere of 5% CO_2_. The medium was changed every 3~4 days. All cells were prepared to use at 37 °C for 24 hours CO_2_ incubation after sub-cultured into new dishes by trypsinization (0.25% trypsin / 0.02% EDTA).

### 3.4. Animals

The Laboratory Animal Ethics Committee of Taipei Medical University approved the study protocol. Wistar rats were purchased from the Center of Experiment Animals, National Taiwan University, Taipei, Taiwan. Rats were housed in plastic cages in a temperature and humidity controlled environment and bred at the Experimental Animal Center of Taipei Medical University. All experiments were performed in accordance with the Animal Experiments of Taipei Medical University guidelines and the general principles for the care and use of laboratory animals as approved by the Chinese Society of Laboratory Animal Sciences, Taiwan. All efforts were made to minimize animal suffering and to reduce the number of animals used.

### 3.5. Preparation of rat brain mitochondria

Rat brain was removed and immediately perfused with ice-cold normal saline and homogenized in chilled phosphate buffer (0.1 M, pH 7.4) containing potassium chloride (1.17%) using a Potter Elvehjem homogenizer. The homogenate was centrifuged at 800 × *g* for 5 min at 4 °C in a refrigerated centrifuge to separate the nuclear debris. The supernatant containing mitochondria were obtained after centrifuging at 10,500 × *g* for 20 min at 4 °C. 

### 3.6. Lactate dehydrogenase (LDH) assay

Membrane damage was assessed by LDH leakage and entered into the culture medium. The LDH activity in the supernatant of cells was determined in an indirect colorimetric assay based on the generation of 1 μmole NADH by reduction of 1 μmole L-lactate, and NADH amount depended on conversion of 2-[4-iodophenyl]-5-phenyltetrazolium chloride (INT) by diaphorase at room temperature [7].

### 3.7. Thiobarbituric acid reactive substances (TBARS) and malondialdehyde (MDA) assay

The assay for brain mitochondria lipid peroxidation (LPO) was performed in accordance with the method of Wong *et al* [21]. The LPO inhibitory activity of γ-mangostin was determined by performing a thiobarbituric acid reactive substances (TBARS) assay, and MDA(TBA)_2_ product was quantitatively analyzed by HPLC. The reaction mixture solution in a total volume of 250 μL containing 50 μL of brain mitochondria, 100 μL of PBS buffer, 50 μL of ferrous ammonium sulfate (Fe(NH_4_)_2_(SO_4_)_2_) solution, and 50 μL of compound was incubated at 37 °C for a period of 60 min. Each reaction was terminated by the addition of 375 μL of H_3_PO_4_, 200 μL of distilled water, and 125 μL 2-thiobarbituric acid. The reaction mixture was incubated at 90 °C for a period of 66 min. The tubes were then placed in an ice bath and 350 μL of methanol-NaOH was added. Each sample was then centrifuged at 13,000 rpm for 5 min at 4 °C whereupon the data was recorded and the inhibition of lipid peroxidation calculated according to the following formula: inhibition (%) = ((A_ control (532 nm)_ - A_ sample (532 nm)_) / (A_ control (532 nm) _- A_ blank (532 nm)_)) × 100.

### 3.8. Cell viability assays

A 3-(4,5-dimethylthiazol-2-yl)-2,5-diphenyltetrazolium bromide (MTT) assay was used to measure cell viability. MTT was reduced to a purple formazan dye by mitochondrial enzymes in the process of actively respiring but not necessarily proliferating cells. Cells were seeded in a 24-well cell culture plate at a density of 3 × 10^5^ cells/mL overnight, whereupon the medium was replaced by γ-mangostin diluted with culture medium (10, 20, 40, 80, 100, and 200 μM). After 24 hours drug treatment, the drug-containing medium was replaced with an equal volume (250 μL) of fresh medium containing MTT and incubated for 4 hours at 37 °C. 0.04 N HCl in isopropanol (250 μL) was then added and the medium was shaken for 30 min. Finally, the optical density (OD) was detected at 600 nm and the cytotoxicity index (CI in %) was calculated according to the equation: CI (%) = [1 - (T/C)] × 100%; where T and C represent the mean OD of the treated (T) and control (C) groups. The half maximal inhibitory concentration (IC_50_) of the cells was then measured [22].

### 3.9. Morphological study using fluorescence microscopy

U87 MG and GBM 8401 cells were seeded at a density of 5 × 10^5^ cells/well into 6-well plates. After 24 hours of adherence, cells were treated with and without a series of concentrations of γ-mangostin (0.5–100 μM) for 24 hours. Giemsa cell staining was conducted before examination by zoom-in 200 fold microscopy.

### 3.10. Apoptosis and intracellular ROS determination

The cells were treated with 80μM γ-mangostin at time intervals of 8, 12, and 24 hours, then washed with ice-cold phosphate-buffered saline (PBS) and fixed in 70 % ethanol at −20 °C for at least 1 hour. After 70% EtOH fixation, cells were washed twice, and incubated in 0.5 mL 0.5% Triton X-100 / PBS at 37 °C for 30 min, and then 1 mg / mL RNaseA was added. Cells were stained with 0.5 mL of 50 μg/mL PI for 10 min. Fluorescence emitted from the propidium-DNA complex was quantitated after excitation of the fluorescent dye by FACScan flow cytometry [23]. DCHDA was used to examine intracellular peroxide levels. The same cells were seeded in a 24-well cell culture plate at a density of 3 × 10^5^ cells/mL and adhered for 24 hours. These two kinds of gliomas cell groups were then treated with H_2_O_2_ (1 mM) and γ-mangostin (80 μM) for 1 hour respectively, and then catalase (CAT, 400 U/mL) was added in order to scavenge the resting H_2_O_2_ for half an hour. Finally, the cells were dyed with DCHDA before being examined by flow cytometry [20].

### 3.11. Detection of the mitochondrial function

DiOC_6_(3) is a fluorescent dye used to detect the function of mitochondria with a decrease in the fluorescent intensity of DiOC_6_(3) being indicative of a loss of mitochondrial membrane potential of the cells. Cells were seeded at a density of 3 × 10^5^ cells /well in 24-well plates and underwent γ-mangostin (80 μM) treatment for 1 hour. In order to evaluate the γ-mangostin induced apoptosis status, the treated cells were then combined with catalase (400 U/mL) or an equal volume of PBS for 3 hours. The cells were dyed with DiOC_6_(3) before being examined by flow cytometry [20].

### 3.12. Statistical analysis

All values for tables and figures were presented as mean ± standard deviation (SD) for three determinations. The significance of the difference from the respective controls for each experimental test condition was assessed using Student’s *t*-test for each paired experiment. A *p* value < 0.01 or < 0.05 was regarded as indicating a significant difference from the control group.

## 4. Conclusions

γ-Mangostin, with an IC_50_ that was significantly lower than the clinical chemotherapeutic agent, BCNU, strongly inhibited glioblastoma multiforme cell proliferation. Our study focused on the mechanism of γ-mangostin-induced growth inhibition in U87 MG and GBM 8401 cells. We observed condensed cells and hypodiploid cells which carry on several intracellular ROS productions and mitochondrial dysfunction in the γ-mangostin treated U87 MG and GBM 8401 cells. It was shown that γ-mangostin may mediate cytotoxicity via apoptosis in U87 MG and GBM 8401 cells.

Nowadays, almost all cancers have a program for clinical therapy, but those for human brain tumors lack the efficacy seen in other forms of cancer. Epidemiological studies have shown that dietary phytochemicals provide beneficial effects in relation to cancer prevention [24,25,26,27]. These findings provide a relevant basis for developing γ-mangostin as an agent for cancer prevention. Notably, a significantly inhibited rate of high-grade glioblastoma growth was observed for the first time. We conclude that γ-mangostin is a strong lead compound candidate in the treatment of brain cancer cell growth, and may directly affect glioblastoma cancer susceptibility through its modulation of cell proliferation and apoptosis.
